# Psychological Problems and Academic Motivation in University Students: A Cross-Sectional Study One Year after the COVID-19 Lockdown in Italy

**DOI:** 10.1007/s11126-025-10138-6

**Published:** 2025-04-03

**Authors:** Alda Troncone, Gaia Caldarelli, Marina Cosenza, Gaetana Affuso, Mariagiulia Sacco, Maria Ciccarelli, Barbara Pizzini

**Affiliations:** 1 Department of Psychology, University of Campania “Luigi Vanvitelli”, Viale Ellittico 31, 81100 Caserta, Italy; 2 Department of Psychology, Telematic University “Giustino Fortunato”, Benevento, Italy

**Keywords:** Psychological distress, Loneliness, Emotion regulation, University students, Academic motivation

## Abstract

This study was aimed at exploring students’ psychological condition (operationalized through psychological distress and perceived loneliness) one year after the COVID-19 lockdown and testing its role in predicting academic motivation. The possible mediating role of emotional regulation in this relationship was also investigated. A non-random sample of university students completed a cross-sectional online survey assessing their psychological distress, feelings of loneliness, emotion regulation strategies, and academic motivation. Structural equation modeling multi-group analysis was used to examine the relation between variables. The final sample comprised 479 students (62.8% female, *M*age = 22.16 ± 2.79 years) from several degree courses attending Italian universities. Of these, 56.6% and 41.5% of the participants showed high levels of psychological distress and perceived loneliness, respectively. Psychological distress and loneliness were positively associated with problems with emotion-regulation abilities and negatively associated with academic motivation. The lack of emotional awareness, in turn, reduced their academic motivation levels. Analysis of the indirect effects showed that psychological distress affected academic motivation via awareness (β = -.02, *p* < .05 and β = -.03, *p* < .05 in males and females, respectively). Overall, the model was able to predict academic motivation in university students, explaining a reasonable percentage of the variance (14% for males and 17% for females). These results indicate a meaningful effect of students’ psychological condition on academic motivation. Implications for the definition and implementation of actions to support students in managing psychological problems and feelings of loneliness and to foster students’ psychological health in university settings are discussed.

**Supplementary Information:**

The online version contains supplementary material available at 10.1007/s11126-025-10138-6.

## Introduction

There is a consensus that university students can be considered a vulnerable group at increased risk of developing psychological problems, and that, in the last few years, the proportion of students with mental health problems has risen [1, 2]. Challenges commonly associated with transitions to adulthood (e.g., lack of a stable income, academic pressure, fear of the future, financial worries experienced while studying, lack of a stable life structure, and increased responsibilities) are deemed to make students’ college years an extremely critical and stressful time [3, 4]. Most university students have, in fact, been described as experiencing stress in several life areas (e.g., in terms of their finances, health, and romantic relationships), and significant associations have been found between stress and the odds of developing mental disorders [5, 6]. Furthermore, a significant finding from the literature for consideration is that age is deemed to be a demographic factor associated with psychological distress and mental health disorders in university students [7, 8].

Unsurprisingly, a large body of evidence from epidemiological studies is available that describes increased psychological problems in university students when compared to the general population [8–10]. Specifically, according to a wide survey recently administered to 19 colleges across eight countries, about 35% of first-year students have reported at least one mental health disorder, with major depressive disorder being the most common (21.2% lifetime prevalence), followed by generalized anxiety disorder (18.6%), panic disorder (5.0%), broad mania (3.5%), and alcohol abuse or dependence (6.8%) [7]. During the COVID-19 pandemic, university students’ mental well-being was extensively tested.

Systematic reviews conducted to estimate the global impact of the COVID-19 pandemic on college students’ mental health have described significantly higher prevalence rates of depression, anxiety, and stress symptoms (depression varying from 26 to 50%, anxiety from 28 to 58%, stress symptoms from 31 to 71%) [11–16] among college students than those observed in the general population during the pandemic [17]. Poor interaction with teachers, colleagues, and friends, or a lack thereof, a troubling learning environment (mainly due to difficulties in adapting to the switch to online learning, and to new assessment methods), worries about their financial situation and the consequent possible economic problems in their families, and worries about the future, were mentioned as the main factors affecting students’ mental health [11–16].

In Italy, starting from the lockdown period (March 9 to May 18, 2020), universities were closed, so that lessons, exams, degrees, and all academic activities were replaced by distance education, provided through online platforms. Italian university students had to wait until September 2021 to be able to physically access their universities and return to their classrooms (on condition that they had an EU Digital COVID Certificate), even though the resource of distance teaching was maintained, and the state of emergency persisted, with all Italian region-specific restrictive measures being based on risk scenarios.

Several studies were conducted on Italian university student samples in order to assess psychological condition aspects, such as subjective well-being, psychopathological symptoms (mainly depression, anxiety, and distress) [18], as well as sleep quality, eating habits and problems [19, 20], emotional states and experiences [21, 22], memory, attention, and concentration during and after the first lockdown [23–25]. Similarly, personal concerns about university studies during the COVID-19 pandemic, distance learning/study difficulties and related matters, resilience skills, and concern and fear resulting from the pandemic were measured [26–28].

However, despite attention being paid to feelings of loneliness in a recent study on college students’ condition during the pandemic [1], in Italian research, there has been no such study focusing on university students’ perception of loneliness during COVID-19. In this regard, it should be noted that loneliness––as a probable result of the prolonged social isolation enacted to combat the pandemic––has recently been recognized as a “critical public health concern” [29].

Because perceived loneliness has proved to be highly related to a wide range of mental and physical health problems [30–33], long before the pandemic provided a specific context, research on young adults’ psychological well-being should take this serious mental phenomenon into account. Similarly, in Italian research, little attention has been given to the relationship between university students’ psychological condition and their academic motivation. This lack of attention is particularly noteworthy given the role of motivation in academic success. According to self-determination theory (SDT), motivation consists of coexisting and complementary forms of behavioral regulation and plays a central role in learning from primary school through to higher education [34]. Unsurprisingly, academic motivation has been found to be related to academic achievement, learning engagement and students’ well-being [34, 35]. It has also been considered a protective factor in students’ intention to drop out of their studies [36].

To the best of our knowledge, in Italian research, only Marzoli et al. (2021) have addressed changes in academic motivation during the transition to online learning, demonstrating a significant decline [22]. In light of the effects that mental health problems have on academic performance and the dropout risk [37–40] and considering that the impact of the COVID-19 pandemic on students’ mental health will probably continue to show its effects for years to come [41, 42], it is important to monitor university students’ psychological condition over time.

Moreover, in examining the relationship between students’ psychological condition and academic motivation, emotion regulation processes deserve particular attention. There is evidence for the transition to university being associated with difficulties in regulating emotions [43, 44]. People with an impaired ability to regulate their emotions have been described as being more susceptible to developing psychopathology [45, 46], and, in university students, emotion regulation difficulties have been found to be the best predictor of maladaptive behavior specifically [47]. Appropriate emotion regulation abilities are deemed to favor more adaptive behaviors for managing negative emotional states [48, 49], such as those resulting from stressful conditions, like a pandemic outbreak. Moreover, evidence suggesting the effects of emotion regulation on academic performance [50–52] led to explore its possible mediating role in the relationship between psychological problems and academic motivation.

Given the above-mentioned considerations, this study aimed to evaluate:

1. University students’ psychological condition (operationalized through psychological distress and perceived loneliness), providing a detailed picture of their psychological state one year after the Italian lockdown.
2. The association between students’ psychological distress, feelings of loneliness, and academic motivation, along with the possible mediating role of emotional regulation strategies.

It was hypothesized that:

1. university students would report high levels of psychological distress and perceived loneliness;
2. a direct-effects model would indicate that psychological distress and loneliness negatively influenced academic motivation; and
3. the effect of psychological condition on academic motivation would be mediated by specific emotion dysregulation dimensions.

Considering the differences found between males and females in terms of psychological distress level and academic motivation [53–56], the moderating role of gender was considered in all expected relationships. In addition, in light of evidence [57, 58] suggesting more problems during the pandemic in first-year university students, age was included as a covariate, potentially influencing all variables of interest. The predictive model of academic motivation was also tested both in more and less psychologically distressed students.

## Methods

### Participants and Procedure

An observational cross-sectional study was carried out. Participants were recruited using a snowball sampling technique among university students attending courses in different disciplinary areas and in different regions in northern, central, and southern Italy, via an online questionnaire. To recruit a large sample, no exclusion criteria were specified.

The sample size was calculated using the Raosoft Sample Size Calculator (Raosoft, Inc., 2004, Seattle, USA, http://www.raosoft.com/samplesize.html). As per the Anagrafe Nazionale Studenti (2021),^1^ in Italy, during the academic years 2020–2021/2022, there were more than 1,800,000 students registered for either Bachelor’s or Master’s degree courses (2020/2021 = 1,839,846; 2021/2022 = 1,822,141). Thus, with a confidence interval of 95% and a margin of error of 5%, the sample size required was calculated as 377. Considering an additional 30% (*n* = 113) of possible dropouts and to increase the study’s reliability, the target sample size was increased to *N* = 490 participants. The survey link was disseminated through the social network and WhatsApp groups the students belonged to, and they were encouraged to pass the survey link to others.

The students, after reading the introduction to, and aims of, the survey, were asked to voluntarily participate, giving informed consent by a specific flag on the first page of the survey. Data were collected from March to December 2021, when university courses in Italy were being provided as a mixed in-presence and remote teaching modality. In particular, the survey was administered during the teaching period (i.e., March to April 2021; November to December 2021) to reduce potential confounders that might increase or decrease psychological problems (i.e., summer holidays). No compensation was given to the participants in the study.

The Helsinki Declaration of Ethical Principles for research involving human participants was followed. The study received the approval of the Ethics Committee of the Department of Psychology of the University of Campania “Luigi Vanvitelli” (Approval no. 08, 09/03/2021).

### Measures

The participants completed an internet-based survey, administered anonymously, implemented using a Google form. The first section of the survey contained questions asking about sociodemographic characteristics (i.e., gender, age), enrollment year, type of university course attended, off-course student condition (i.e., if all exams within the prescribed period of time had been passed), and whether they had had any previous or current contact with psychological or psychiatric university (or out-of-university) mental health services. The second section consisted of four scales that measured the variables of interest.

#### Psychological Problems

The Clinical Outcomes in Routine Evaluation––Outcome Measure (CORE–OM) [59, 60], originally designed to evaluate the outcome of psychological treatment, is a 34-item self-report measure for assessing the level of psychological distress/problems. The items are rated on a 5-point Likert-type scale (ranging from 0 = not at all, to 4 = most or all of the time), referring to individual experiences that occurred over the last week, and covering four domains––subjective well-being (four items assessing feelings about the self and optimism about the future), problems/symptoms (12 items assessing depression, anxiety, physical problems, and trauma), life/social functioning (12 items assessing general daily functioning, and close and social relationships), and risk (six items assessing risk to self and to others). In all the domains, higher scores indicated more distress and problems. The CORE–OM represents a reliable and valid instrument for measuring symptoms, problems, and impairment in psychological functioning, showing solid psychometric properties [61–63]. The Italian version of the CORE-OM has been shown to display strong reliability coefficients and satisfactory construct and criterion validity [64].

In order to distinguish between clinical and non-clinical cut-off values, the four dimensions (scales for both males and females) indicated in the original validation and the Italian version [63, 64] were considered. In the present study, the internal consistency of the instrument was found to be satisfactory, yielding a Cronbach’s alpha above 0.70 for each CORE-OM dimension (well-being = 0.762; problems/symptoms = 0.901; life/social functioning = 0.809; risk = 0.717; total score = 0.937). Corrected item–total correlation (CITC) values were also considered acceptable, with items ranging between 0.32 and 0.76 (Table S1) (except for items 3, 6, 19, 22, and 34, which had CITC values < 0.3).

#### Feelings of Loneliness

The University of California–Los Angeles (UCLA) Loneliness Scale version 3 (LS3) [65] is a 20-item self-report measure of an individual’s subjective feelings of loneliness. The items (11 loneliness and nine non-loneliness) are rated on a 4-point Likert scale (from 1 = never to 4 = always). Higher scores indicate greater loneliness. Generally, the UCLA-LS3 has been shown to display strong psychometric properties—with satisfactory internal consistency, test–retest reliability, ample criterion, concurrent and construct validity—which support the instrument’s reliability and validity as a measure of loneliness across various ethnicities and languages [66–68]. For the present study, the UCLA–LS3 Italian-validated version was used [69]. In this study, the UCLA–LS3 showed excellent internal consistency (Cronbach’s alpha = 0.931). The CITC values for each item were greater than 0.3 (range: 0.314–0.757), indicating high reliability for questionnaire measurement (Table S1). In line with published findings, a UCLA–LS3 total score of ≥ 47 was considered as indicating higher than normal level of loneliness [29, 70, 71].

#### Emotion Regulation

The Difficulty in Emotion Regulation Scale (DERS) [72] is a 36-item multi-dimensional self-report measure of emotion dysregulation and emotion self-regulation strategies. The items are rated on a 5-point Likert-type scale (from 1 = almost never to 5 = almost always) and refer to six subscales––non-acceptance of emotional responses (non-acceptance); difficulties engaging in goal-directed behaviors when distressed (goals); impulse control difficulties when distressed (impulse); lack of emotional awareness or inattention to emotional responses (awareness); limited access to emotion regulation strategies (strategies); and lack of emotional clarity (clarity). The total scores range from 36 to 180, with higher scores indicating more difficulty in the emotion regulation component measured. The DERS has shown good psychometric properties, including strong reliability and adequate construct and predictive validity [72]. The Italian version of the DERS has also displayed high internal consistency and good concurrent validity indicating that this instrument can be considered a useful tool for measuring emotional regulation strategies in the Italian context [73, 74]. In the present study, good internal consistency values were found (Cronbach’s alpha: non-acceptance = 0.904; goals = 0.874; impulse = 0.869; awareness = 0.811; strategies = 0.898; clarity = 0.874; and total score = 0.880). CITC values ranged between 0.411 and 0.812, indicating high reliability for questionnaire measurement (Table S1).

#### Academic Motivation

The Academic Motivation Scale (AMS) [75] is a 20-item self-report questionnaire designed to evaluate the regulation of motivation based on the self-determination theory [76]. Academic motivation is conceptualized according to the degree of autonomy on which the individual behavior is based, expressed through a continuum of increasing self-determination, from not self-determined (amotivation pole) to self-determined (autonomous regulation pole) behavior [77], from being externally motivated to becoming internally and autonomously driven to perform certain behaviors. The items are rated on a 5-point Likert scale (0 = does not correspond at all to 4 = corresponds exactly) and distributed in five subscales––amotivation, external regulation, introjected regulation, identified regulation, and intrinsic motivation. A high score for each subscale indicates higher endorsement of that motivation type. In addition, the Relative Autonomy Index (RAI) was computed, based on Vallerand and Ratelle’s (2002) indications [78]. High RAI scores indicate a higher degree of self-determined perceived academic motivation and autonomy.

The AMS has demonstrated adequate reliability and validity among university students [79]. In the present study, the Italian 20-item version of the AMS was adopted [80], which has demonstrated satisfactory psychometric properties, including high internal consistency and good predictive validity. In this study, the AMS showed good reliability, as indicated both by the Cronbach’s alpha coefficients for each AMS dimension that exceeded 0.7 (amotivation = 0.825; external regulation = 0.918; introjected regulation = 0.746; identified regulation = 0.934; intrinsic motivation = 0.904) and by the CITC values that ranged between 0.329 and 0.882 (Table S1).

### Statistical Analyses

All variables are presented as frequencies, percentages, and the mean ± SD. Multiple-group structural equation modeling (SEM), including observed variables, was used to test the hypotheses, with gender as the grouping variable. The variables included in the model were: psychological distress^2^ and loneliness perception as independent variables; all subdimensions of emotion-regulation ability as mediators; and academic motivation (RAI) as a dependent variable. Age was included as a covariate influencing all the other variables. An additional model that analyzed the relationship between loneliness perception and academic motivation through emotion-regulation ability was also tested, with less and more psychologically distressed students as the grouping variable. In this last model, age and gender were included as covariates influencing all the other variables.

To test the equivalence of the structural parameters across the groups (male vs. female; more vs. less psychologically distressed students), the parameters were freely estimated in the first step. In the second step, structural paths and correlations were constrained to be equal across groups. The analyses were conducted using maximum likelihood estimations [81]. The indirect effects were calculated using the indirect-effect test implemented in MPLUS 7.4 software. As for fit indices, the Comparative Fit Index (CFI) [82], the Tucker–Lewis Index (TLI) [83], and the root mean square error of approximation (RMSEA) [84] were taken into consideration, with the following values used to accept the model: CFI and TLI ≥ 0.90 and RMSEA ≤ 0.08 [85]. Finally, the Satorra–Bentler chi-square difference test (ΔSBχ^2^) was also performed to compare the fit of the nested models [86]. To check the adequacy of the sample size in the SEM, the rule of having between five and 10 observations for each estimated parameter was adopted [87].

Descriptive and bivariate analyses were performed using SPSS version 21.0 software for Macintosh, and the SEM were computed using Mplus version 7.4 software.

## Results

### Students’ Psychological Condition

A convenience sample of *N* = 490 students, attending Italian universities across several different degree courses, was recruited. Of these, *n* = 11 participants were excluded due to incomplete or erroneous completion of the questionnaire. The distribution of the final sample (*N* = 479) characteristics (e.g., sex, university degree, and area of study) of the CORE–OM and UCLA–LS3 scores are reported in Table 1.

**Table 1:** Socio-demographic characteristics of the sample

	Whole sample *N* = 479
Gender (female) (*n*, %)	301 (62.8)
Age (years, month) *M(SD)*	22.16 (2.79)
Territorial areas (*n, %*)^a^	
North	50 (10.4)
Center	34 (7.1)
South	382 (79.8)
Degree level (*n*, %)	
Bachelor	277 (57.8)
Master	105 (21.9)
5/6-years courses	97 (20.3)
Course (*n*, %)	
Medicine	41 (8.6)
Psychology	19 (4)
Art and humanities	121 (25.3)
Political sciences	52 (10.9)
Law	40 (8.4)
Engineering	86 (18)
Architecture	10 (2.1)
Economics	45 (9.4)
Nursing	12 (2.5)
Biology	32 (6.7)
Other^b^	21 (4.4)
Off-course student (yes) (*n*, %)	61 (12.7)
Student worker (yes) (*n*, %)	139 (29)
Previous contacts with own university mental health service (yes) (*n*, %)	63 (13.2)
Previous contacts with mental health service (yes) (*n*, %)	6 (1.3)
CORE-OM	
Subjective well-being/ ≥ cut-off (*n*, %)	1.83 (.89)/ 266 (55.5)
Symptoms/ ≥ cut-off (*n*, %)	1.62 (.84)/276 (57.6)
Life functioning/ ≥ cut-off (*n*, %)	1.37 (.62)/238 (49.7)
Risk/ ≥ cut-off (*n*, %)	.2 (.39)/ 118 (24.6)
Total score/ ≥ cut-off (*n*, %)	1.3 (.61)/ 271 (56.6)
UCLA Total score/ ≥ cut-off (*n*, %)	44.79 (10.69)/199 (41.5)

Data are presented as mean values and standard deviations unless otherwise stated

^a^*n* = 13 missing data; ^b^ university courses not classified in previous categories

As provided in Table 1, 56.6% of the students showed a high level of psychological distress, as indicated by the CORE–OM total score ≥ clinical cut-off value. Specifically, about 55.5% of the students reported low subjective well-being, 57.6% a high level of problems/symptoms (e.g., anxiety, stress, physical problems, and sleeping difficulties), 49.7% problems in daily life functioning, and in close and social relationships. Moreover, about 24.6% of the students reported scores above the clinical risk cut-off point (risk of self-harm or doing harm to others). Almost half (41.5%) of the participants showed high loneliness levels, as indicated by the UCLA–LS3 total score ≥ cut-off value.

### Psychological Condition, Emotion Regulation Abilities, and Academic Motivation

The Pearson correlations among the study variables were computed separately for the males and females, and these are provided in Table 2.

**Table 2:** Correlations among the variables

	1	2	3	4	5	6	7	8	9
1. Psychological distress	-	.64***	.53***	.52***	.50***	.17**	.69***	.55***	-.27***
2. Perceived loneliness	.61***	-	.40***	.31***	.30***	.27***	.50***	.52***	-.29***
3. Non acceptance	.57***	.54***	-	.54***	.59***	.25***	.73***	.50***	-.07
4. Goals	.58***	.35***	.50***	-	.57***	.03	.71***	.42***	-.10
5. Impulse	.39***	.27***	.59***	.46***	-	.13*	.70***	.46***	-.12*
6. Awareness	.31***	.14	.17*	.10	.17*	-	.17**	.44***	-.23***
7. Strategies	0.70***	.57***	.73***	.68***	.60***	.25***	-	.58***	-.13*
8. Clarity	.59***	.44***	.53***	.40***	.44***	.49***	.61***	-	-.24***
9. Academic motivation	-.38***	-.34***	-.25***	-.27***	-.17*	-.26***	-.31***	-.26***	-

Correlations for males are below the diagonal, correlations for females are above the diagonal

*p < .05; ** *p* < .01; *** *p* < .001

The predictive model of psychological distress and loneliness perception on academic motivation through emotion-regulation ability was tested**.** The fit indices of the unconstrained model were χ^2^ (0) = 0, *p* = 0.0, RMSEA = 0.0 (0.0; 0.0), TLI = 1, and CFI = 1 (saturated model). During the second step of the analysis, the structural paths and correlations were constrained to be equal across genders. The fit indices for the constrained model were χ^2^ (45) = 55.04, *p* = 0.15, RMSEA = 0.03 (0.0; 0.05), TLI = 0.99, and CFI = 0.99. The Δχ^2^ statistic showed that the fit of the constrained model across genders was significantly better than the fit of the unconstrained model, Δχ^2^ (45) = 55.04, *p* > 0.05. Therefore, the constrained model with total invariance across groups was adopted. The sample of 479 subjects met the requirements for ensuring the stability and reliability of the implemented model estimates by having at least five observations available for the 81 estimated parameters.

**Fig. 1:**
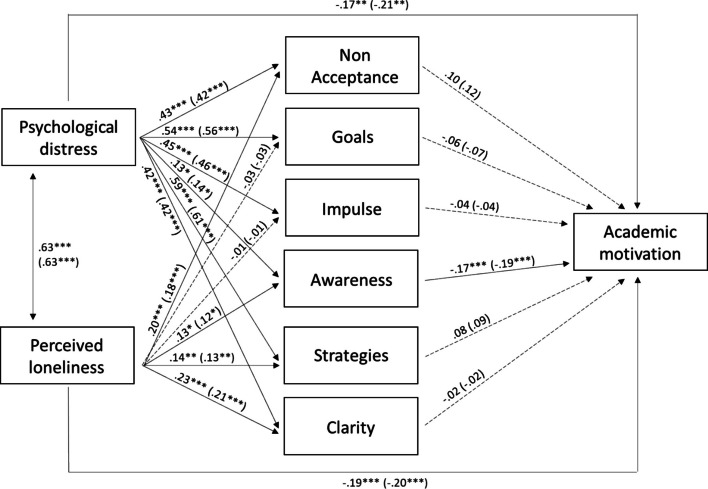
Psychological distress, loneliness, emotion regulation and academic motivation. *Note*. Relations between psychological distress, loneliness perception, all subdimensions of emotion-regulation ability and academic motivation. Standardized path coefficients. Parameters for males are shown without brackets, parameters for females are shown in brackets. *p < 0.05 **p < 0.01 ***p < 0.001. To simplify, the paths from age and the correlations between all subdimensions of emotion-regulation ability were reported in the text

As shown in Fig. 1, the results indicated that: psychological distress was positively correlated with loneliness perception; psychological distress was positively associated with all subdimensions of emotion regulation ability and negatively associated with academic motivation; loneliness perception was positively associated with non-acceptance, awareness, strategies, and clarity, and negatively associated with academic motivation; and awareness was negatively associated with academic motivation. The analysis of the indirect effects showed that psychological distress affected academic motivation via awareness (β = −0.02, *p* < 0.05 and β = −0.03, *p* < 0.05 for males and females, respectively). Age was negatively associated with awareness (β = −0.14, *p* < 0.05 and β = −0.09, *p* < 0.05 for males and females, respectively), strategies (β = −0.11, *p* < 0.01 and β = −0.07, *p* < 0.01), and clarity (β = −0.14, *p* < 0.01 and β = −0.09, *p* < 0.01). All correlations between the subdimensions of emotion regulation ability were significant at *p* < 0.05, except for correlations between awareness and goals, awareness and impulses, and awareness and strategies. Overall, the model explained a reasonable percentage of the variance for academic motivation (14% for males and 17% for females).

The predictive model of loneliness perception on academic motivation through emotion regulation ability was also tested by the more and less psychologically distressed students**.** The fit indices of the unconstrained model were χ^2^ (0) = 0, *p* = 0.0, RMSEA = 0.0 (0.0; 0.0), TLI = 1, and CFI = 1 (saturated model). During the second step of the analysis, structural paths and correlations were constrained to be equal across the less and more psychologically distressed students. The fit indices for the constrained model were χ^2^ (45) = 79.40, *p* < 0.01, RMSEA = 0.06 (0.03; 0.08), TLI = 0.97, CFI = 0.94. The delta chi-square statistics showed that the fit of the constrained model was significantly worse across groups than the unconstrained model [Δχ^2^ (45) = 79.40, *p* < 0.01]. The examination of the modification indices suggested that we could improve the fit of this model by estimating the correlation between strategies and non-acceptance, strategies and goals, and strategies and impulse freely across groups. After this refinement, the model fitted the data well: χ^2^ (42) = 55.63, *p* > 0.05, RMSEA = 0.04 (0.0; 0.06), TLI = 0.99, CFI = 0.98). Therefore, we chose this last model, with partial invariance across groups, because it showed better fit and a non-significant delta chi-square statistic [Δχ^2^ (42) = 55.63, *p* > 0.05]. The sample of 479 subjects met the requirements for ensuring the stability and reliability of the implemented model estimates by having at least five observations available for the 88 estimated parameters.

**Fig. 2:**
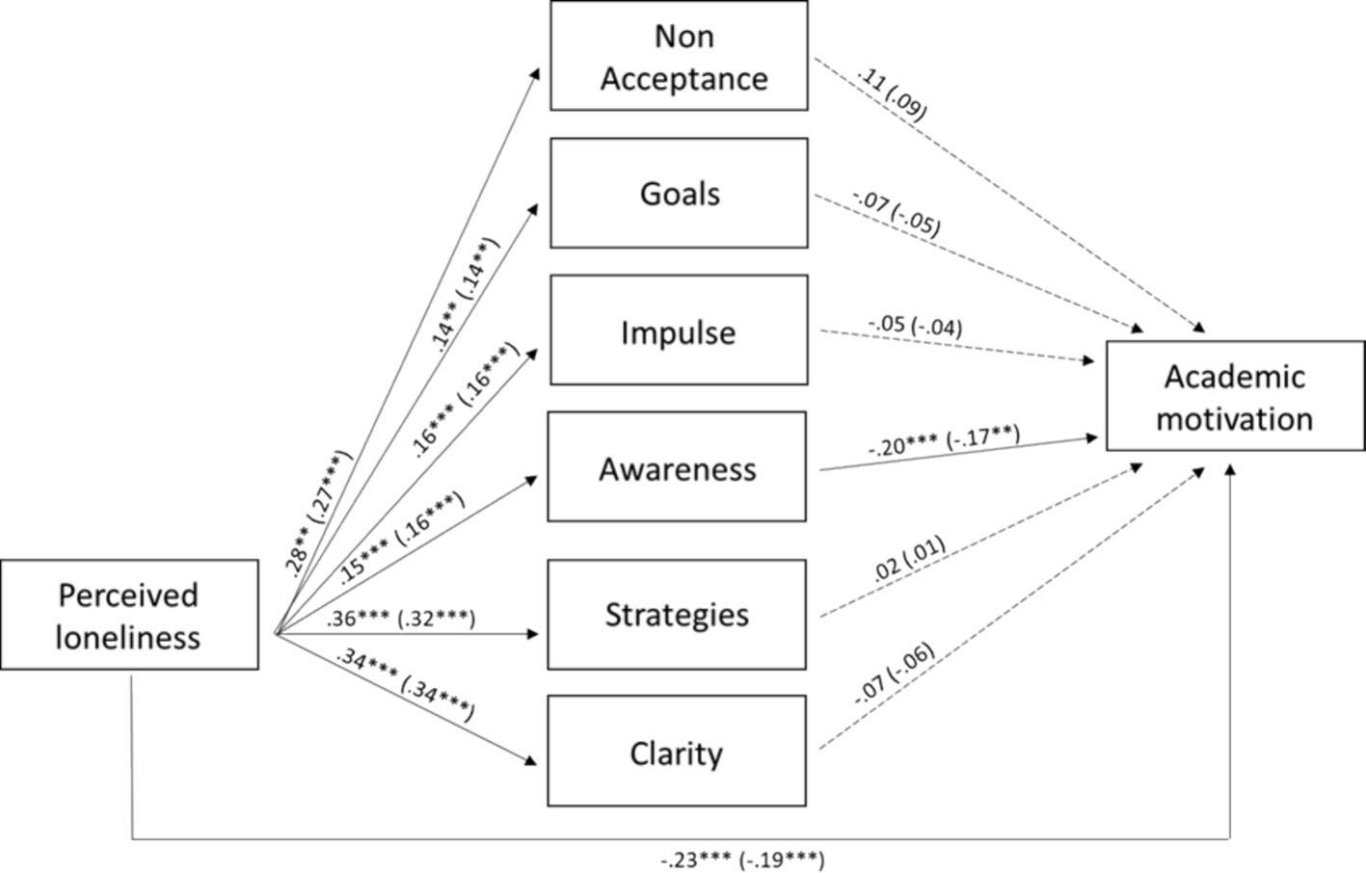
Loneliness, emotion regulation and academic motivation in higher and lower psychological distressed students. *Note*. Relations between loneliness perception, all subdimensions of emotion-regulation ability and academic motivation. Standardized path coefficients. Parameters for lower psychological distressed students are shown without brackets, parameters for higher psychological distressed students are shown in brackets. *p < 0.05 **p < 0.01 ***p < 0.001. To simplify, the paths from age and gender and the correlations between all subdimensions of emotion-regulation ability were reported in the text

As shown in Fig. 2, the results indicated that: loneliness perception was positively associated with all subdimensions of emotion regulation ability and negatively associated with academic motivation; and awareness was negatively associated with academic motivation. The analysis of the indirect effects showed a significant indirect effect of loneliness perception on academic motivation via awareness (β = −0.03, *p* < 0.05 for less and more psychologically distressed students). Age was negatively associated with awareness (β = −0.13, *p* < 0.05 and β = −0.10, *p* < 0.05 for less and more psychologically distressed students, respectively), strategies (β = −0.12, p < 0.05 and β = −0.08, *p* < 0.05), and clarity (β = −0.14, p < 0.01 and β = −0.11, *p* < 0.01). Gender (1 = males and 2 = females) was positively associated with non-acceptance (β = 0.15, *p* < 0.01 and β = 0.13, *p* < 0.01 for less and more psychologically distressed students, respectively), impulse (β = 0.10, *p* < 0.05 and β = 0.09, *p* < 0.05), stimulation (β = 0.14, *p* < 0.01 and β = 0.11, *p* < 0.01), and academic motivation (β = 0.19, *p* < 0.001 and β = 0.14, *p* < 0.001). All correlations between the subdimensions of emotion regulation ability were significant at *p* < 0.05, except for correlations between awareness and goals, awareness and impulses, and awareness and strategies. Overall, the model explained a reasonable percentage of the variance for academic motivation (15% for less psychologically distressed students and 10% for more psychological distressed students).

## Discussion

This observational study, for the first time, aimed to investigate the presence of psychological distress and perceived loneliness levels one year after the Italian lockdown, and the interplay between these factors and academic motivation in Italian university students, considering the potential mediating role of emotional regulation abilities.

Our findings provided support for all three hypotheses formulated: the university students reported high levels of psychological distress and perceived loneliness; both psychological distress and loneliness negatively influenced their academic motivation; and the effect of psychological condition on academic motivation was mediated by specific emotion dysregulation dimensions.

Regarding the first hypothesis, the present results indicated that, one year after the Italian lockdown, a considerable percentage of the students showed high general psychological distress. Specifically, the students who scored above the CORE–OM cut-off values for all dimensions considered ranged from 24.6% (risk subscale) to 56.6% (total score). Compared with the proportion of participants who exceeded the cut-off points of the CORE–OM measures in previous studies, these rates were higher than the prevalence data collected from a non-clinical university student sample (i.e., 29%: Bewick et al., 2008 [88]; 47.4%: Iturrioz et al., 2018 [89]).

Similarly, 41.5% of the students perceived themselves as socially isolated. In particular, the students reported mean levels of loneliness (*M* = 44.79) that were slightly higher than those found in prior studies carried-out during the pandemic on the general population (*M* = 43.8: Killgore et al., 2020 [29]), but slightly lower than those yielded by other studies recently carried out on university students (*M*range = 48.3–51.7: American College Health Association, 2022 [1]; *M* = 51.13: Karami et al., 2020 [90]; *M* = 49.1: Pretorius, 2022 [91]).

This level of psychological distress is considered to be in line with the high prevalence of psychological problems and psychopathological symptoms described in studies on university students from before the COVID-19 pandemic. In this regard, the psychological burden resulting from the life transition associated with students’ college years and the task implied (e.g., changes in life conditions, academic and parental pressures, the challenge of dealing with a new educational and social context, excessively high expectations about future careers) means students’ university years have frequently been described as an extremely stressful life period [6, 92, 93]. In addition, the pandemic added further challenges, such that the drastic changes associated with the physical, social, and academic environment further exacerbated university students’ psychological condition (e.g., the dramatic decrease in face-to-face social interaction, and the mirrored necessity of spending a lot of time staring at a screen, as inevitable consequence of the online learning system) [11–14, 16].

Similarly, the high levels of perceived social isolation reported here can be considered, on one hand, to be the expected manifestation of the experience of loneliness already described in university students (e.g., as a result of their young age, being away from the family home, and having to develop new friendships at university) and, on the other hand, as a common experience due to the lack of human interaction imposed by the COVID-19 restriction measures [29, 94].

Our results also confirmed the second hypothesis, because psychological distress and perceived loneliness were found to be negatively associated with academic motivation. These relationships remained significant even after controlling for the confounding effects of age, regardless of gender. These results are consistent not only with previous literature that emphasized the effect of both mental health problems and perceived social isolation on academic motivation and functioning [37, 39, 95, 96], but also with data indicating significant associations between psychological distress, disability, and lower academic achievement [40]. More recently, Giusti et al. (2021) [23] identified learning concentration impairment, anxiety about COVID-19 contagion, and depressive symptomatology as the strongest predictors of poor academic performance during distance education in Italian university students evaluated a few months after the start of the lockdown.

Unsurprisingly, the predictive model also showed that psychological distress was positively correlated with perceived loneliness, confirming evidence from previous studies on university students who had reported that the feeling of social isolation was associated with anxiety, depressive symptoms, and general mental health problems [97, 98].

Finally, regarding the third hypothesis, as expected, the effect of psychological condition on academic motivation was mediated by emotion dysregulation. The present results suggested, in fact, that psychological distress and loneliness may increase the lack of awareness of university students’ own emotions (i.e., the presence of difficulties in identifying the specific emotion one is experiencing), which in turn reduced academic motivation levels. In addition, the analysis of the indirect effects showed that psychological distress is associated with academic motivation via awareness. Moreover, the direct effect of perceived loneliness on academic motivation, and indirectly as mediated by awareness, remained unchanged in the less and more psychologically distressed students, seeming to suggest a key role of feelings of social isolation on motivation, regardless of the presence and degree of psychological problems.

Consistent with the present results, studies on university students have recently described emotion regulation difficulties as significantly correlated with psychological distress, pathological personality dimensions, and emotional disorders [99, 100], and associated with academic procrastination [51] and predictors of maladaptive impulsive behaviors [47]. Furthermore, amotivation (a subcomponent of academic motivation according to SDT) has been found to be associated with difficulties in emotional regulation among university students [52]. Similarly, along with the evidence indicating loneliness being associated with poor mental health and academic performance [30, 101], previous research has indicated that loneliness is also related to emotion regulation strategies [102, 103] suggesting that difficulties in emotion awareness are an important factor in understanding the development of loneliness, especially in individuals with psychopathological symptoms [104].

### Limitations

This study had several limitations. First, the lack of random selection and the possible selection bias related to voluntary and convenience recruitment limited the external validity and the generalizability of the results. Second, the use of self-report instruments to measure variables of interest may have affected the accuracy in collecting the data. Moreover, the narrow period during which data collection occurred, as well as its heterogeneity in terms of the characteristics of academic life (the participants completed the survey over months of suspended face-to-face teaching, when they could not even access university facilities, and months of combined classroom and distance learning, when only certificate-holders could enter the university) and in terms of different stressful periods in the students’ life (e.g., exams), limited this study’s provision of a true and exhaustive overview of the psychological condition of university students one year after the pandemic. Third, the cross-sectional design of the study did not allow any causal inferences about the relationships between the main variables to be deduced. Additionally, not accounting for potential sources of heterogeneity in participants’ characteristics (e.g., previous contact with mental health services) may have resulted in overlooking important subgroup effects.

To obtain a more comprehensive overview of the topic under investigation, these limitations should be addressed in future research by employing more robust methodologies. For instance, utilizing a nationally representative sample chosen via random sampling techniques and assessed using different methods (e.g., questionnaires and semi-structured clinical interview assessments) may help to establish more conclusive findings. Similarly, since the impact of COVID-19 on mental health can be long-lasting, enduring beyond the acute or subacute stages that occur during the first months following a pandemic [105, 106], further studies should explore psychological problems over time. To do so, future research may benefit from adopting longitudinal investigations to examine the long-term psychological consequences of the pandemic and confirm the presence and nature of the relationship between psychological problems and academic motivation. Examining these relationships over time is necessary to fully understand their causal nature and determine the trajectory and directionality of these changes over time. This would enable the identification of key predictors of academic motivation and make causal inferences more feasible.

### Implications

Several implications were revealed by this research. First, by showing, for the first time, the role of emotion awareness ability in mediating the effects of psychological problems and perceived loneliness (indifferently across more and less psychologically distressed groups) on academic motivation, a significant theoretical contribution was added to the existing literature examining how psychological disease in university students is related to academic outcomes.

Additionally, these findings, which provide an overview, albeit not definitive, of the symptoms and problems that students experienced during this challenging time have important implications for the delivery of clinical psychological mental health services in university settings. To begin with, the high prevalence of psychological problems found in university students suggests the importance of ensuring resource allocation and targeting effective care and interventions. Specifically, where lacking, establishing a psychological service within universities is imperative to deliver prompt psychological assistance. Because the present results suggest that young adults’ academic motivation is significantly related to the presence of consistent psychological problems and strong negative emotions and thoughts related to the experience of loneliness, the Italian university system is warmly invited to further appropriate and tailored psychological interventions, in order to improve university students’ psychological well-being and, consequently, academic performance. That is, different types of interventions, including consultation and psychotherapy, should be made available according to students’ psychological needs and the severity of their problems. Considering the high level of loneliness that we found, which may have been caused or exacerbated by the social restrictions imposed during the pandemic, it may be important to deliver interventions and plan activities that can promote mental health and provide opportunities (e.g., through involvement in peer groups) for lonely individuals to have social contact with others beyond those in virtual environments utilizing digital technologies [107].

In addition, as our results suggest, emotion regulation processes, especially deficit in the capacity to recognize one’s own affective states, may be a suitable target variable for psychological clinical preventive and treatment interventions in university settings, in order to reduce the impact of mental distress on academic motivation, and, once again, performance objectives. Furthermore, since existing barriers and stigma toward mental health issues and help-seeking behavior [108] may influence how youth manage and approach psychological problems, increasing resistance or delaying requests for psychological help, it is essential to promote mental health literacy in educational contexts.

## Conclusions

Although our results did not demonstrate any causal relationships between the main variables, they did confirm the key role of mental-health support in university contexts to meet university students’ psychological needs. Considering that high levels of psychological distress increase academic failure and dropout risk [109], that suicidal behavior seems to be more common in students who report greater psychological distress [93], and that there is a relationship between academic motivation and risk of dropout [110, 111], efforts to support university students’ psychological needs are a necessity. This perspective is also consistent with SDT, which suggests that supporting students' basic psychological needs would increase their psychological wellbeing, academic motivation, and engagement, which in turn would improve their academic performance [34]. Importantly, the present results suggest that special attention should be paid to young adults’ feelings of loneliness, and to academic motivation, regardless of psychological distress levels. To reduce perceived social isolation, improve and strengthen individual abilities to successfully deal with psychological burdens related to both their emerging adulthood phase and to their pandemic challenges, actions can be implemented and specifically directed toward more vulnerable students.

Our findings provide suggestions about the opportunity to develop and implement plans that address the whole university community in order to reduce psychological distress and feelings of loneliness, and consequentially reduce the likelihood of university students’ intention to leave university courses before completing their studies.

Similarly, preventive and treatment psychological interventions can be implemented and directed toward students affected by psychopathological problems. Specifically, dedicated assistance and appropriate mental treatment could be offered to students facing high psychological distress in order to support them in their academic studies. As indicated by the present study, emotional dysregulation is a key individual vulnerability factor that can lead to young adults’ psychological distress and loneliness affecting their academic motivation. This should be considered when planning psychological preventive interventions as a way of curbing academic motivation decrease.

To reduce the global academic burden and prevent the risk of dropout, academic agencies should ensure adequate resource allocation and offer free psychological consultation services for the early detection of psychological distress, while also promoting public campaigns that destigmatize mental suffering, thereby facilitating access to these services.

## Supplementary Information

Below is the link to the electronic supplementary material.

Supplementary file1 (DOCX 24 KB)

## Data Availability

Data is available upon reasonable request.

## Footnotes

1 Anagrafe Nazionale Studenti (2021). Retrieved July 20, 2021, from https://anagrafe.miur.it/index.php

2 It was decided to consider psychological distress as a full scale because the objectives of the study were to analyze the association between general levels of distress (rather than specific components), loneliness, and academic motivation one year after the COVID-19 lockdown, and to test its role in predicting academic motivation. In contrast, the need to test the possible mediating role of emotional regulation (as assessed by the DERS) led us to consider the subscales of the DERS.
