# Supplementary material for: Psychological Problems and Academic Motivation in University Students: A Cross-Sectional Study One Year after the COVID-19 Lockdown in Italy
Source: Psychiatr Q. 2025 Apr 3;96(3):683–704. doi: 10.1007/s11126-025-10138-6 (PMC12460576; doi:10.1007/s11126-025-10138-6)
Supplement: Supplementary file 1 — Supplementary file1 (DOCX 24 KB) [file 11126_2025_10138_MOESM1_ESM.docx]

**Supplementary TABLE S1**. Item details, examples, subscales, and reliability indices for each study measure

| Measure (construct measured) | Item type | Subscales | Example of item | Total N of items | % of test | Cronbach’s Alpha | CITC range |
| --- | --- | --- | --- | --- | --- | --- | --- |
| CORE-OM  (Psychological distress) | 5-points on Likert scale (from 0= not at all to 4= most or all the time) | Subjective Well-Being | I have felt O.K. about myself | 4 | 3.64 | .762 | 0.528-0.624 |
|  |  | Problems/ Symptoms | I have felt tense, anxious or nervous | 12 | 10.91 | .901 | 0.450-0.709 |
|  |  | Life/Social Functioning | I have felt terribly alone and isolated | 12 | 10.91 | .809 | 0.233-0.603 |
|  |  | Risk to self and others | I have been physically violent to others | 6 | 5.45 | .717 | 0.238 - 0.696 |
|  |  | TOT |  | 34 | 30.91 | .937 | 0.12-0.76 |
|  |  |  |  |  |  |  |  |
| UCLA-LS3  (Perceived loneliness) | 4-points Likert scale (from 1 = never to 4 = always) | - | How often do you feel you lack companionship? | 20 |  | .931 | 0.314-0.757 |
|  |  |  |  |  |  |  |  |
| DERS  (Emotion regulation strategies) | 5-points Likert scale (from 1 = almost never to 5 = almost always) | Non-Acceptance | When I’m upset, I become angry with myself for feeling that way | 6 | 5.45 | .904 | 0.621-0.810 |
|  |  | Goals | When I’m upset, I have difficulty getting work done | 5 | 4.55 | .874 | 0.559-0.803 |
|  |  | Impulse | I experience my emotions as overwhelming and out of control | 6 | 5.45 | .869 | 0.411-0.812 |
|  |  | Awareness | I pay attention to how I feel | 6 | 5.45 | .811 | 0.456-0.718 |
|  |  | Strategies | When I’m upset, I believe that I will remain that way for a long time | 8 | 7.27 | .898 | 0.496-0.770 |
|  |  | Clarity | I am clear about my feelings | 5 | 4.55 | .874 | 0.596-0.788 |
|  |  | TOT |  | 36 | 32.73 | .880 | 0.411-0.812 |
|  |  |  |  |  |  |  |  |
| AMS  (Academic motivation) | 5-points Likert scale (from 0 = does not correspond at all to 4 = corresponds exactly) | A-motivation | Honestly, I don’t know; I really feel that I am wasting my time | 4 | 3.64 | .825 | 0.631-0.735 |
|  |  | External regulation | Because it is important for what I decided to do in the future | 4 | 3.64 | .918 | 0.752-0.857 |
|  |  | Introjected regulation | To prove that I can succeed in this | 4 | 3.64 | .746 | 0.329-0.686 |
|  |  | Identified regulation | Because it is useful to achieve my goals in life | 4 | 3.64 | .934 | 0.781-0.882 |
|  |  | Internal regulation | Because it is good to learn new things in this area | 4 | 3.64 | .904 | 0.713-0.874 |
|  |  | TOT |  | 20 | 18.18 | .705 | 0.392-0.882 |
|  |  |  |  | 110 | 100 |  |  |

*Note*: CITC Corrected item-total correlation; CORE-OM Clinical Outcomes in Routine Evaluation––Outcome Measure; UCLA-LS3 University of California–Los Angeles Loneliness Scale version 3; DERS Difficulty in Emotion Regulation Scale; AMS Academic Motivation Scale
